# Current knowledge of idiopathic scoliosis among practising physiotherapists in South Africa

**DOI:** 10.4102/sajp.v76i1.1500

**Published:** 2020-11-09

**Authors:** Abraham du Toit, Nassib Tawa, Dominique C. Leibbrandt, Josette Bettany-Saltikov, Quinette A. Louw

**Affiliations:** 1 Division of Physiotherapy, Faculty of Medicine and Health Sciences, Stellenbosch University, Cape Town, South Africa; 2 Department of Health and Social Care, Teesside University, Middlesbrough, United Kingdom; 3 Center for Research in Spinal Health & Rehabilitation Medicine, Department of Physiotherapy, College of Health Sciences, Jomo Kenyatta University of Agriculture and Technology, Nairobi, Kenya

**Keywords:** idiopathic scoliosis, physiotherapy, knowledge, bracing, treatment, causes, screening, diagnosis, survey

## Abstract

**Background:**

Idiopathic scoliosis (IS) is a common musculoskeletal condition with a multi-factorial aetiology characterised by a three-dimensional torsional deformity of the spine.

**Objectives:**

To ascertain the current level of knowledge on IS among registered practising physiotherapists who expressed an interest in orthopaedic, muscular, manual and manipulative therapy in South Africa (SA).

**Method:**

An online survey was used to collect the data. The questions were based on an existing questionnaire, validated by a South African panel of experts in the field of musculoskeletal physiotherapy and updated based on the 2016 Society of Scoliosis Orthopaedic Rehabilitation Treatment (SOSORT) guidelines for the assessment and management of IS.

**Results:**

Two hundred and twenty-three physiotherapists spread across the nine provinces of SA met the inclusion criteria and were included in our study. Our findings showed that about one-third (33.6%) of the physiotherapists could answer more than 50% of these questions correctly, and 16.5% could answer 70% of the questions correctly in relation to the widely accepted guidelines on IS management.

**Conclusion:**

The participants had a poor understanding of the diagnosis and treatment involved in managing patients with IS and a lack of knowledge regarding the methods of conservative treatment for scoliosis. Future studies should be aimed at assessing intervention strategies to improve the knowledge of IS in physiotherapists in SA, especially regarding diagnosis and identifying appropriate management strategies.

**Clinical implications:**

Physiotherapists are often the first contact practitioners for patients presenting with scoliosis and therefore need to have the necessary clinical knowledge on the assessment and management of IS. Our study can improve the awareness among the South African physiotherapists regarding IS and its complex presentation and management.

## Introduction

Scoliosis is a three-dimensional torsional deformity of the spine and trunk measuring ≥ 10 degrees as measured by using the Cobb angle method, affecting humans from infancy to after puberty (Negrini et al. 2012). The major physical changes that are associated with this ‘musculoskeletal condition’ are shoulder tilt (one shoulder higher than the other), asymmetrical waistline and an elevation of one side of the back or ‘rib hump’ (Negrini et al. 2018).

The exact cause of idiopathic scoliosis (IS) remains unknown (Burger et al. 2019), and several factors have been implicated in the aetiology, including structural elements of the spine, endocrine function, genetic transmission, changes associated with the ‘growth spurt’ during adolescence and hormones associated with the onset of puberty (Burger et al. 2019). Idiopathic scoliosis presents in two clinical subtypes, structural or functional curves (Sarnadskiy 2012). Structural scoliosis curves are fixed, and they may result in one or more compensatory non-structural curves. Clinically, these types of curves are progressive, and therefore these cannot be abolished as one or more segments of the spine possess a fixed lateral curve. On the other hand, functional curves are non-progressive and fully correctable as they are caused by modifiable factors such as poor posture, disc herniation and leg length inequality (Sarnadskiy 2012).

The global prevalence of IS is estimated at 2% – 3% of the country’s population; however, the rates of prevalence and severity are higher in girls than in boys (Meirick et al. 2019). Africa, in particular, has a paucity of documented reports and inconsistent findings on the prevalence of IS, with limited school-based epidemiological studies conducted in Nigeria, Rwanda, Ghana and South Africa (SA) (Adegoke, Akinpelu & Taylor 2011; Bello et al. 2016; M’Kumbuzi et al. 2019; Van Rensburg 2006). These school-based epidemiological studies report prevalence values ranging from 3.3% to 55% in school-age children.

The current conventional management options available for IS include a range of conservative approaches and sometimes surgery. However, the evidence-based practice guidelines by the Society of Scoliosis Orthopaedic Rehabilitation Treatment (SOSORT), published in 2018, recommend a comprehensive conservative management for IS with curves ≤ 19° (Negrini et al. 2018).

The goals of the comprehensive conservative management are to stop curve progression at puberty (or even reduce it), to prevent or treat respiratory dysfunction, to prevent or treat spinal pain syndromes and to improve aesthetics via postural correction (Negrini et al. 2018). Therefore, adequate and up-to-date knowledge regarding screening, diagnosis, classification and treatment of IS among practising physiotherapists is critical for the implementation of the best available evidence to guide orthopaedic rehabilitation in patients with IS.

A few studies have investigated the present knowledge of IS among physiotherapy students in the United States of America, the United Kingdom and Poland, and the results indicate a rather poor level of knowledge (Black et al. 2017; Ciazynski, Czernicki & Durmala 2008; Drake, Glidewell & Thomas 2014). To date there has never been a study conducted to assess the basic knowledge of IS among practising physiotherapists in any African country.

Studies have shown that cases of adolescent idiopathic scoliosis (AIS) that are undiagnosed could lead to major debilitating trunk deformity, pain in adulthood, pulmonary complications and, in extreme cases, mortality (Kenner, McGrath & Woodland 2019). Physiotherapists who are often the first contact clinicians, and who implement physiotherapeutic scoliosis-specific exercises (PSSE) in the management of patients with scoliosis, can have a positive influence on the course of scoliosis. Compared with other ‘more serious’ health problems such as human immunodeficiency virus (HIV)/acquired immune deficiency syndrome (AIDS), diabetes, obesity, heart disease and cancer, IS has received a low priority in the healthcare systems globally (Negrini et al. 2018), and SA is no exception.

Thus the aim of our study was to ascertain the current level of knowledge on IS among registered practising physiotherapists in SA. Our study evaluates and describes the basic knowledge of physiotherapists (in the fields of orthopaedic, neuromuscular or manipulative therapy) regarding the screening, diagnosis and conservative treatment of patients with IS. Our study also aimed to increase awareness among the included South African physiotherapists about IS and its complex presentation and management and to identify knowledge gaps regarding IS, that need to be addressed.

### Contribution to field

It is an essential requirement for a physiotherapist to be proficient in the management of musculoskeletal-related conditions such as scoliosis and other spinal deformities. In SA, registered physiotherapists have first-line practitioner status, which means that the public does not require a referral to be seen by a physiotherapist, and self-referral to physiotherapy in SA is becoming increasingly common particularly in the private sector (Diener 2016). Therefore, the likelihood of a physiotherapist being the first contact practitioner for a patient presenting with scoliosis is high. It is important that practising physiotherapists have the necessary knowledge and clinical understanding on the screening and recognition of signs and symptoms suggestive of IS. Because IS is a progressive disorder, early detection and identification are very important in improving patient outcomes. If AIS is detected early, it can lead to better decision-making regarding the course of conservative treatment and whether surgery can be avoided (Canavese & Kaelin 2011).

The three previous studies conducted in Poland, the United States of America and the United Kingdom that assessed the knowledge of IS among physiotherapy students concluded that the knowledge was unacceptable (Black et al. 2017; Ciazynski et al. 2008; Drake et al. 2014). To date and to the authors’ knowledge, there has never been a study conducted to assess the basic knowledge of IS among practising physiotherapists in SA.

## Methodology

A quantitative descriptive study was used to collect data through an online survey that targeted physiotherapists who were registered with the Health Professions Council of South Africa (HPCSA), Orthopaedic Manipulative Physiotherapy Group (OMPTG) and the South African Society of Physiotherapy (SASP). Therefore, practising physiotherapists registered with the above groups comprised the study population. Registered physiotherapists, younger than 75 years of age, who are registered with the HPCSA as independent practitioners actively practising physiotherapy in any of the nine provinces of SA, were included and were required to have clinical experience and an interest in the areas of orthopaedic, muscular, manual or manipulative therapy. Undergraduate physiotherapy students were excluded because of their limited clinical experience.

The study population was a total of 1361 South African physiotherapists who were all members of the OMPTG, and physiotherapists who were not members of the OMPTG but who expressed an interest in orthopaedic and musculoskeletal physiotherapy. We sampled across all provinces, and all physiotherapists who met the inclusion criteria were invited to participate. Recruitment of the OMPTG and non-OMPTG was conducted based on the SASP registration database. Two hundred and thirty-seven physiotherapists completed the online survey. Nine physiotherapy students and three physiotherapists who indicated that they were not interested in the orthopaedic, muscular, manual or manipulative therapy/management of clients were excluded as they did not meet the inclusion criteria. A further two physiotherapists (one OMPTG member and one non-OMPTG member) completed the questionnaire after the cut-off date (31 July 2019) and were therefore excluded from our study.

## Procedure

### Questionnaire development

Firstly, we aimed to identify an initial survey questionnaire that had been used in previous studies. We opted to use a questionnaire by Drake et al. (2014) after obtaining permission from the authors. This questionnaire was developed by Drake et al. (2014) by using a theoretical framework from a previously completed questionnaire by Ciazynski et al. (2008) and utilising the information provided within the 2011 SOSORT guidelines (Negrini et al. 2012). This questionnaire was selected for use in our study because it was the most suitable as it tested the knowledge of IS in physiotherapy students. This questionnaire is available as Online Appendix 1.

Secondly, we validated this questionnaire and updated as necessary to conduct a survey on SA physiotherapists. The initial questionnaire (Drake et al. 2014) was reviewed and validated by a panel of three experienced researchers and university lecturers in the field of musculoskeletal physiotherapy in SA (ID, EK and CV). Face validity was established, and the panel confirmed that the same questionnaire would provide an overview of the knowledge on IS among physiotherapists in SA. The survey was then updated and five additional questions (Online Appendix 2) aimed at investigating the physiotherapist’s confidence in the screening, assessment, management and education of a scoliosis patient were included as suggested by the latest 2016 SOSORT guidelines (Negrini et al. 2018). The final questions included in the survey are presented in Table 1. These questions assessed different aspects of IS and were divided into the following categories: definition, cause, development, prevalence, diagnosis, treatment, bracing and the familiarity with types of conservative treatments for IS.

**TABLE 1:** Questions and categories included in the final questionnaire.

Category	Question
Definition	What is idiopathic scoliosis?
Cause	What causes idiopathic scoliosis?
Development	When does idiopathic scoliosis commonly develop?
Prevalence	How prevalent is idiopathic scoliosis among scoliosis patients?
Diagnosis	How is the diagnosis of idiopathic scoliosis commonly confirmed?
Treatment	The treatment of idiopathic scoliosis using therapeutic exercise should include?
Bracing	When is bracing recommended for patients with idiopathic scoliosis?
Familiarity	What method of conservative treatment of idiopathic scoliosis are you most familiar with?
Evidence-based research	According to evidence-based research, what has proven to be the most effective form of conservative management in idiopathic scoliosis?
Screening	Would you feel confident using Adam’s forward bend test and the Scoliometer?
Educational support/confidence	Would you feel confident providing educational support to a client presenting with idiopathic scoliosis?
Management confidence	Would you feel confident in the management of a client with idiopathic scoliosis?
Opinion	Do you feel scoliosis specific physiotherapy exercise interventions can be beneficial in the management of idiopathic scoliosis?

IS, idiopathic scoliosis.

### Data collection and analysis

The reviewed and validated questionnaire, which consisted of 15 multiple choice questions, was transcribed onto an online platform, and the survey monkey program was used to present the information and capture all the responses. The online questionnaire was accessible to physiotherapists for a period of 5 weeks in June to July 2019, at which time all the data from the survey monkey platform were collated for analysis.

Descriptive statistics (percentages) were applied to describe the demographics of the participants as well as the responses for each question.

To assess for differences in the knowledge on screening, education and exercise prescription of self-referring patients with IS among OMPTG physiotherapists and non-OMPTG physiotherapists, Chi-squared tests were used to compare the proportions for every response to every question. The categorical variables were also analysed by using Chi-squared tests to further compare the proportions between the OMPTG members and the non-OMPTG members at a 95% level of significance.

### Ethical consideration

This study was approved by the Health Research Ethics Committee of Stellenbosch University (S18/04/079), and the study procedures conformed to the South African Medical Research Council (MRC) Ethical Guidelines for Research.

## Results

### Demographic characteristics of study participants

Two hundred and twenty-three physiotherapists from across the nine provinces of SA participated in our study. The provinces of Gauteng (34.5%) and the Western Cape (28.3%) had the majority of the participants. One hundred and sixteen (52%) of these physiotherapists are members of the OMPTG, and the other 107 (48%) are not members of the OMPTG but indicated on the questionnaire that they were interested in orthopaedic and musculoskeletal physiotherapy (Table 2). About half (53%) of all the participants had postgraduate qualifications. More of the participants from the OMPTG (67%) and 37% in the non-OMPTG had a postgraduate qualification.

**TABLE 2:** Years of clinical experience in the included sample (*n* = 223).

Variable	≤ 3 years (%)	4–9 years (%)	10–20 years (%)	> 20 years (%)
Years of clinical experience	21.5	23.8	33.6	21.1
OMPTG	11.2	20.7	42.2	25.9
Non-OMPTG	32.7	27.1	24.3	15.9

OMPTG, orthopaedic manipulative physiotherapy group; Non-OMPTG, non-orthopaedic manipulative physiotherapy group.

### Definition of idiopathic scoliosis

The question (What is IS?) assessed whether the physiotherapist was aware of scoliosis being a three-dimensional torsional deformity of the spine. A total of 108 (48%) answered the question correctly and 91 (41%) incorrectly thought that IS is a lateral curvature of the spine; 18 (8%) suggested that it is a two-dimensional deformity of the spine whilst seven (3%) were unsure.

### Cause of idiopathic scoliosis

**FIGURE 1:**
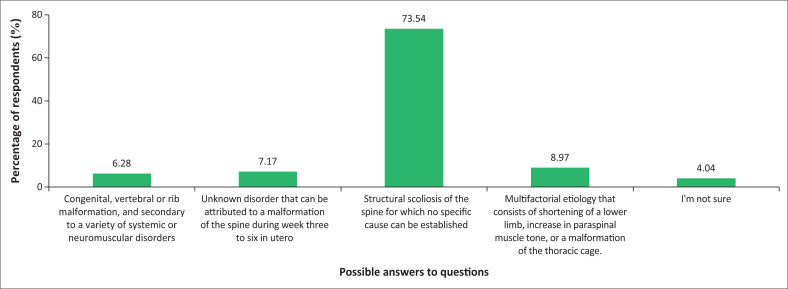
Knowledge on causes of idiopathic scoliosis (*n* = 223).

When assessed whether the physiotherapists understood the aetiology of IS, most of them, 163 (73.54%), answered the question correctly and a small proportion, nine (4.04%), of the participants were unsure. Figure 1 illustrates the various responses by the study participants.

### Prevalence of idiopathic scoliosis

A total of 35 (15.7%) of the physiotherapists (*n* = 223) who were included in the study answered the question (How prevalent is IS amongst patients with scoliosis?) correctly. The majority, 130 (58.3%), indicated that they were not sure and selected this answer for the prevalence question (Table 1).

### Diagnosis of idiopathic scoliosis

**FIGURE 2:**
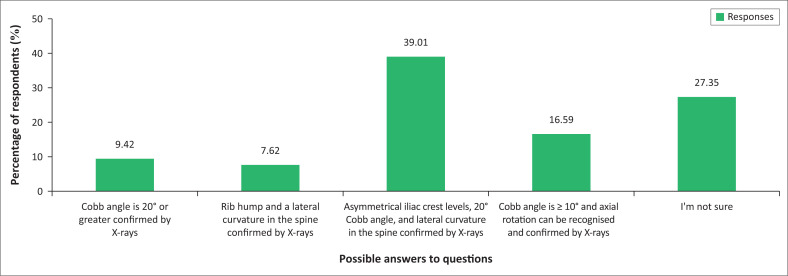
Diagnosis of idiopathic scoliosis (*n* = 223).

A total of 37 (16.59%) answered this question correctly (Figure 2). It is concerning that a large proportion, 87 (39.01%), incorrectly suggested that the diagnosis is based in the iliac crest levels. Sixty-one (27.35%) indicated that they were not sure about the diagnostic criteria for IS.

### Treatment of idiopathic scoliosis

**FIGURE 3:**
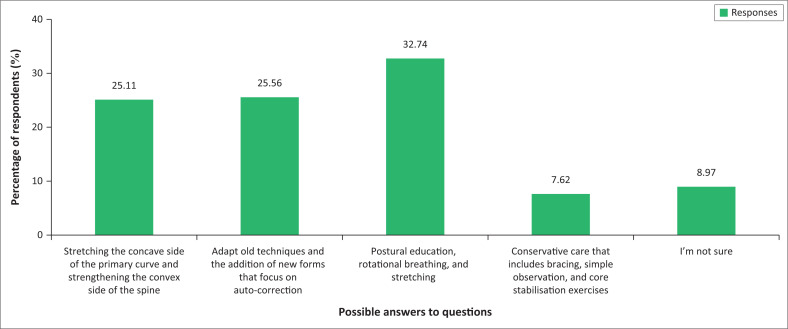
Treatment of idiopathic scoliosis (*n* = 223).

About one-quarter (25.56%) answered this question correctly. Seventy-four (33%) thought that the treatment of IS by using therapeutic exercise should include ‘postural education, rotational breathing, and stretching, shown to be the gold standard in research when considering treatment of IS’. A further 56 (25%) said that treatment should include ‘focus on stretching the concave side of the primary curve and strengthening the convex side of the primary curve in the spine’. Figure 3 indicates the various responses to the question about the treatment of IS (see Table 1).

### Bracing of idiopathic scoliosis

**FIGURE 4:**
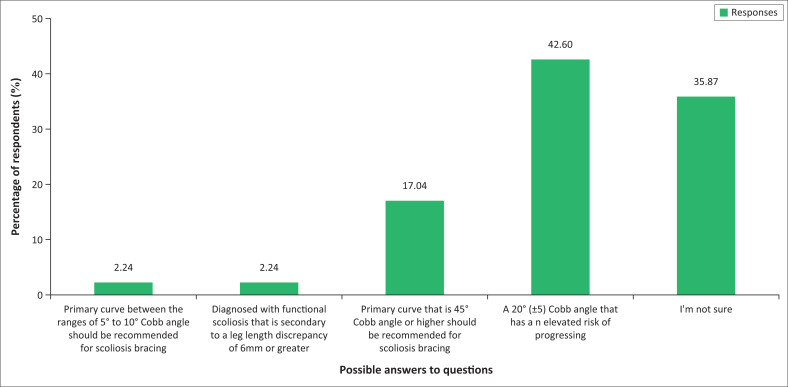
Recommendation for bracing of a patient with idiopathic scoliosis (*n* = 223).

The results show that a total of 95 (42%) (*n* = 223) answered this question correctly by indicating that bracing should be recommended for patients with IS when the curve is 20° (± 5) Cobb angle, and also when the patient has an elevated risk of progression. However, it is alarming that 80 (35.87%) indicated that they were unsure, whilst just over one-fifth of the participants gave an incorrect response. The responses are illustrated in Figure 4.

### Familiarity with conservative treatment methods for idiopathic scoliosis

The question ‘What method of conservative treatment of IS are you most familiar with?’ was aimed at identifying how familiar the physiotherapists were with the different types of conservative treatment for IS. Although there are numerous PSSE schools and methods with published evidence of efficacy in the conservative treatment of IS (Negrini et al. 2018:3), many physiotherapists in our study failed to recognise any of the methods. One hundred and seventy (76%) indicated that they were not sure. Ninety (78%) of the physiotherapists in the OMPTG indicated that they were not sure compared with 79 (74%) in the non-OMPTG. The answers to this question also had an option where the participants could enter a treatment method that they were familiar with in case that method was not listed in the available options, but this yielded no responses.

### Differences between the orthopaedic manipulative physiotherapy group and non-orthopaedic manipulative physiotherapy group

The differences in the responses between OMPTG physiotherapists and non-OMPTG physiotherapists are summarised in Table 3. Chi-squared tests were used to compare the proportions of correct responses between the OMPTG members and the non-OMPTG members at a 95% level of significance. Significantly more participants in the OMPTG identified the definition and diagnosis of IS correctly compared with those in the non-OMPTG.

**TABLE 3:** Differences and percentage of correct responses between orthopaedic manipulative physiotherapy group and non-orthopaedic manipulative physiotherapy group (Chi square test) (*n* = 223).

Variables	Correct response (%)	Incorrect response (%)
**Definition of IS (*p* = 0.01)**		
OMPTG	56.0	44.0
Non-OMPTG	40.0	60.0
**Cause of IS (*p* = 0.60)**		
OMPTG	75.0	25.0
Non-OMPTG	72.0	28.0
**Prevalence of IS (*p* = 0.93)**		
OMPTG	15.5	84.5
Non-OMPTG	15.9	84.1
**Diagnosis of IS (*p* = 0.001)**		
OMPTG	25.0	75.0
Non-OMPTG	8.0	92.0
**Treatment of IS (*p* = 0.38)**		
OMPTG	28.0	72.0
Non-OMPTG	23.0	77.0

IS, idiopathic scoliosis; OMPTG, orthopaedic manipulative physiotherapy group; Non-OMPTG, non-orthopaedic manipulative physiotherapy group.

### Educational support for the client

The question ‘Would you feel confident in providing educational support to a client presenting with IS?’ aimed to assess the confidence of the physiotherapist in educating a client presenting with IS (Table 1). A total of 106 (47.5%) indicated that they would be confident in providing educational support to an IS client, whilst 74 (33.2%) indicated that they would not be confident and 43 (19.3%) indicated they were unsure. The OMPTG and the non-OMPTG again had similar results, with the OMPTG being slightly more confident with a total of 57 (49.1%) compared with the 49 (45.8%) of the non-OMPTG. Thirty-eight (32.8%) of the OMPTG indicated that they were not confident with providing educational support compared with the 36 (33.6%) of the non-OMPTG.

### Screening by using the Adams forward bend test and a scoliometer

The question ‘Would you feel confident evaluating IS using the Adam’s forward bending test and the scoliometer?’ measured the confidence of the physiotherapist when using the Adams forward bend test and the scoliometer in the assessment of an IS client. Close to 80% of the physiotherapists would not be confident using these tools; 110 (49.3%) indicated that they would not be confident and 72 (32.3%) were unsure on how to use these assessment tools. Therefore, only 41 (18.4%) were confident using the Adams forward bend test and the scoliometer. The OMPTG and the non-OMPTG had similar results, with the OMPTG being slightly more confident with a total of 23 (19.8%) compared with 18 (16.8%) of the non-OMPTG.

## Discussion

The main objective of our study was to assess the level of knowledge on selected aspects of IS amongst registered practising physiotherapists with an interest in orthopaedic and musculoskeletal physiotherapy in SA and a proxy measure of physiotherapists who would likely have some experience and knowledge working with clients with IS. The key findings were that the physiotherapists generally had a good understanding of the cause of IS, but relatively less knowledge about the prevalence, diagnosis and evidence-based management for patients with IS. In addition, they were not very familiar with the methods of conservative treatment, education and using assessment tools such as a scoliometer. A large proportion of the participants (76%) were unaware of the presence of various scoliosis schools globally and unable to recognise any treatment methods used for scoliosis rehabilitation. In addition, fewer than 50% of the participating physiotherapists were able to diagnose and define IS, or to determine when bracing would be recommended for patients with IS.

Based on the results, it appears that physiotherapists in SA are ill-equipped to assess and manage patients with IS, and they are unlikely to provide the recommended evidence-based care required to manage such a progressive and time-sensitive condition. This may be due to a relatively low level of exposure of IS, lack of adequate coverage of the topic in undergraduate curricula or limited continuing professional development (CPD) opportunities.

Our findings are similar to the majority of studies conducted in other countries, indicating a low level of basic knowledge of IS amongst physiotherapists (Black et al. 2017; Ciazynski et al. 2008; Drake et al. 2014). Unfortunately, all previous studies have focussed on students and not qualified physiotherapists. The student studies showed low levels of knowledge amongst students in the United Kingdom, the United States of America and Poland (Black et al. 2017; Ciazynski et al. 2008; Drake et al. 2014). However, in our study, the practising physiotherapists performed better than the physiotherapy students in the United Kingdom (Black et al. 2017), where only 7% of the participants answered more than 50% of the questions correctly (Black et al. 2017), and better than the physiotherapy students in the United States of America (Drake et al. 2014), where only 8% of the participants answered 70% of the questions correctly.

The Polish study (Ciazynski et al. 2008) was conducted on a small group (*n* = 37) of students attending the third year of the first degree in physiotherapy. The students had credits in kinaesiotherapy, including methods of conservative treatment of IS according to the 2006 SOSORT guidelines (Kotwicki et al. 2009). These students achieved far superior results regarding familiarity of conservative treatment approaches, compared with the students in two other studies (Black et al. 2017; Drake et al. 2004), with 95% of participants able to identify at least one method of conservative treatment. This suggests that physiotherapists who are educated in line with the SOSORT guidelines are more familiar with scoliosis in addition to the treatment approaches available for this patient group. However, it remains unclear how much of the information will be retained after graduation and especially if these physiotherapists are not regularly exposed to clients with IS.

Our study included qualified physiotherapists, and therefore a direct comparison with physiotherapy students’ levels of knowledge may not be appropriate, although low levels of knowledge were reported in most of the student studies as well as in our study which involved qualified physiotherapists. In addition, our participants had between 10 and 20 years of post-graduate experience and therefore would be expected to perform better because of their clinical experience and practice knowledge. Our findings suggest that there are few physiotherapists in SA with the necessary skills and knowledge to appropriately manage clients with IS using evidence-based approaches. They indicate the need for regular CPD training opportunities to address these knowledge gaps and ensure that clients with IS receive appropriate management and outcomes are optimised.

The level of knowledge about the prevalence of IS and the criteria involved in diagnosing IS were poor. As participants did not understand these two aspects, they subsequently performed poorly in identifying the conservative treatment involved in treating patients with IS. These three areas form an integral part in the management of patients with IS and therefore need to be addressed in the South African physiotherapy community that works in orthopaedics, in particular, those dealing with patients with scoliosis.

The inability to accurately diagnose clients with IS will have a detrimental impact on their prognosis, and therefore physiotherapists who have first-line practitioner status need to be able to both screen and understand the accurate diagnosis of IS. The Society of Scoliosis Orthopaedic Rehabilitation Treatment has indicated that 80% of all scoliosis cases are idiopathic and that this is a diagnosis of exclusion which can only be applied with confidence when other causes of spinal deformity have been eliminated (Meirick et al. 2019). Because of IS being a progressive disorder, early recognition and identification of any individual at risk are extremely important in the rehabilitation of the client and may prevent surgery (Negrini et al. 2018). The Society of Scoliosis Orthopaedic Rehabilitation Treatment supports the conservative treatment of all spinal deformities, and PSSE is one of these conservative therapeutic exercise treatment interventions (Negrini et al. 2018). According to the PSSE principles, each therapeutic exercise method should aim to treat all aspects of the 3D scoliosis deformity and focus on auto-correction in three dimensions to prevent or limit progression (Berdishevsky et al. 2016).

The OMPTG participants had significantly better knowledge than the non-OMPTG ones. In 85% of the questions, the OMPTG performed better than the non-OMPTG, with the most significant differences being in the definition and diagnosis of IS. The OMPTG is a special interest group in SA that focusses on assessing and treating orthopaedic and musculoskeletal conditions. Therefore, one would expect members of this group to be more knowledgeable when it comes to IS patient care and management and are more likely to encounter a patient with IS in their physiotherapy practice. The differences in the number of years of post-graduate experience between the two groups could potentially have had an influence on the responses as the OMPTG had a higher percentage of participants with more than 10 years of post-graduate experience. However, there were no significant differences between groups with regard to conservative treatment involved with IS, and the overall correct responses from both groups were poor. Furthermore, when questioned on the familiarity of the different methods of conservative treatment of IS, the OMPTG was more unsure with 78% failing to recognise any method. Therefore, the OMPTG cannot currently be recognised as the authority on the management and care of patients with IS.

Our study may assist in creating awareness amongst physiotherapists with an interest in orthopaedic and musculoskeletal physiotherapy regarding IS and its complex presentation and management. This can stimulate and potentially aid in government contribution to public healthcare, brace funding and professional school screening services for IS patients.

### Strengths and limitations of the study

This was the first study to examine the current knowledge of IS amongst practising physiotherapists in SA. Our study sample was drawn from the OMPTG and other physiotherapists with an interest in orthopaedic and musculoskeletal physiotherapy and thus represent a group of physiotherapists in SA who will most likely have experience in managing clients with IS. The participant eligibility criteria were thus appropriate for our study, thereby increasing the validity of the results. Moreover, the survey questions are standardised and based on the SOSORT guidelines (2011 and 2016). Because similar questionnaires were used for published international studies, it allowed for comparison among studies.

Our study had a bigger sample size (*n* = 223) than other studies investigating knowledge of IS ranging from 32 to 206 participants (Black et al. 2017; Ciazynski et al. 2008; Drake et al. 2014). It included physiotherapists with a range of experience and specialised interests from all nine provinces in SA, whereas the other studies only investigated the knowledge of a limited sample of student physiotherapists.

The survey provides an overview but not an in-depth understanding of IS knowledge. Assessing IS knowledge is complex and can potentially be improved with face-to-face discussions and interviews, course examination or testing. The information in our study was self-reported and therefore could have been biased, which could have impacted on the outcome.

The physiotherapists who agreed to participate may be those who have a greater interest in improving individual practice and knowledge, and they may also be more knowledgeable about musculoskeletal conditions. The fact that the questionnaires were sent to the participants meant that they had time to do research on IS and prepare their answers although the findings do not suggest that this was the case.

Recruitment was conducted by using the SASP registration database, and no random sampling was undertaken. This could have introduced sampling bias. Future studies should consider using the HPCSA registration list, as this would provide a larger and more diverse potential sample, although we maintain that physiotherapists with an interest or experience in orthopaedics are most appropriate. Future studies could attempt randomisation for selecting participants to improve the generalisability of the findings to all SA physiotherapists.

### Future research

Future research is needed to address the shortcomings in knowledge of IS amongst physiotherapists. Initiatives to address this issue could include the establishment of a national IS network, CPD as well as other online strategies to encourage discussion and drive knowledge acquisition on IS amongst South African physiotherapists and potentially other professions involved in the management of IS as well. The effect of these interventions should be explored in future studies.

## Conclusion

Idiopathic scoliosis is a complex musculoskeletal disorder with an unconfirmed multi-factorial aetiology. Physiotherapists in SA are currently ill-equipped to provide this first point of care for IS as they are not familiar with assessment tools, do not have adequate confidence to educate clients with IS and do not have adequate knowledge of the diagnosis, indications for bracing and evidence-based advice. The findings of our study may create awareness amongst the physiotherapy community and indicate the need for increasing knowledge to improve the outcome of individuals with IS. Initiatives to address these knowledge gaps such as curriculum revision and CPD opportunities are needed. The effects of these strategies should be assessed in future studies.
